# Governance of Public-Private Partnerships for Primary Healthcare in Low- and Lower-Middle-Income Countries, 2000-2023: A Systematic Review

**DOI:** 10.34172/ijhpm.8442

**Published:** 2025-03-08

**Authors:** Georgina Dove, Adam Craig, Ben Harris-Roxas, Angela Kelly-Hanku

**Affiliations:** ^1^University of New South Wales, Sydney, NSW, Australia.; ^2^University of Queensland, Brisbane, QLD, Australia.; ^3^Papua New Guinea Institute of Medical Research, Goroka, Papua New Guinea.

**Keywords:** Governance, Public-Private Partnership, Primary Healthcare, Collaboration, LMIC

## Abstract

**Background:**

Public-private partnerships (PPPs) in the health sector are established to achieve health outcomes by maximising the combined resources of both public and private sectors. Good governance is core to PPP function and success. This paper explores the factors that enable and constrain governance in the delivery of PPPs for primary healthcare (PHC) in low- and lower-middle-income countries (LLMICs).

**Methods:**

A systematic search of four literature databases was conducted to identify peer reviewed articles published between 2000 and 2023 related to the governance of PPPs for PHC in LLMICs. A deductive analysis of data extracted from selected articles against the domains of Greer’s TAPIC (transparency, accountability, participation, integrity, and policy capacity) governance framework was conducted to identify commonly reported enabling and constraining factors.

**Results:**

Of the 4290 records screened, 14 were included. Common enabling factors for governance within each domain of the framework were found: Transparency: unequal and top-down resource allocation, and opaque and resource allocation was a barrier to PPP governance; Accountability and policy capacity: monitoring and evaluation; Participation: partner engagement, covering topics of developing and managing relationships, collaborative activities, and communication; and Integrity: the design of the PPP, covering formal agreements between partners, level of policy direction, and integration within the broader health system.

**Conclusion:**

The five domains of the TAPIC governance framework provide guidance for considering governance in PPPs. The enabling factors identified in the review help facilitate the successful implementation of a PPP and thus influence the PPP’s impact on health outcomes, through establishing and maintaining healthy working relationships between partners, and defining and documenting systems and processes.

## Background

 Public-private partnerships (PPPs) in the health sector are established to achieve health outcomes by maximising the combined resources of both public and private stakeholders.^1,2^ Reich defined the three core features of PPPs as “at least one private for-profit organisation and at least one not-for-profit or public organisation,” “shared objectives for the creation of social value,” and “shared efforts and benefits.”^3^ At the global level, examples of PPPs used to support improved health outcomes include the Global Fund to Fight AIDS, Tuberculosis, and Malaria; and Gavi, the Vaccine Alliance.^4,5^ At the country and local levels, PPPs have been implemented to improve specific health and social outcomes; for example, a health service improvement partnership in Papua New Guinea.^6,7^ Since the 1990s when global health PPPs were increasingly documented,^8,9^ PPPs have become an established and recognised way of working in the health sector by governments in both low- and lower-middle-income countries (LLMICs) and high-income countries.^10^

 In some LLMICs, PPPs are considered “key structures for the definition, evaluation and delivery of many healthcare services.”^11^ PPPs have been used to deliver primary healthcare (PHC) services when resources are scarce and/or different stakeholders can provide different skills and resources,^1^ and to address health system challenges in achieving PHC goals and universal health coverage, such as workforce shortages.^12^ As key mechanisms for the delivery of PHC, PPPs require robust governance to realise their intended outcomes and impact.

 Governance has many definitions, and in this paper we adopt Barbazza and Tello’s explanation that “the governance function characterizes a set of processes (customs, policies or laws) that are formally or informally applied to distribute responsibility or accountability among actors of a given [health] system.”^13^ The authors further delineate the values of governance for example “good governance,” compared to the descriptions and types of governance arrangements such as how relationships are structured to perform functions of governance.^13^ This latter, descriptive perspective of governance enables a wide application of governance concepts, including to governance arrangements in health service delivery partnerships implemented at a local level. Further, this perspective distinguishes the concepts from the commonly used term health governance, which often refers to a national health system or the role and work of international organisations such as the World Health Organization (WHO) in leadership and stewardship of health systems.^13,14^

 While research on the delivery of PHC through PPPs is available,^1,10-12,15^ the governance of locally implemented PPPs for PHC in LLMICs has not been extensively explored. The objective of this paper is to address this gap by exploring governance in PPPs for PHC that are implemented at a local level in LLMIC settings, in recognition of the large number of collaborations that occur at sub-national levels and for service delivery.

## Methods

 A systematic search was conducted to identify peer reviewed literature relevant to the governance of locally implemented PPP for PHC in LLMICs. The review was conducted in line with the Preferred Reporting Items for Systematic Reviews and Meta-Analyses (PRISMA) guidelines.^16^ The PRISMA checklist is provided in Supplementary file 1.

###  Definitions

 A broad definition of PPPs was used, where “public” refers to any government sector organisation (eg, a Ministry of Health), and “private” refers to any non-public sector organisation, including private enterprise and non-governmental organisations (NGOs). This definition was adopted to ensure that literature (and lessons) from a broad range of PPPs was captured. The WHO’s definitions of PHC and governance were used: “[PHC is] a whole-of-government and whole-of-society approach to health that combines three core components: multisectoral policy and action; empowered people and communities; and primary care and essential public health functions as the core of integrated health services”^17^ and “[governance is] ensuring strategic policy frameworks exist and are combined with effective oversight, coalition-building, the provision of appropriate regulations and incentives, attention to system-design, and accountability.”^14^ This WHO definition is aligned with Barbazza and Tello’s definition noted earlier in the paper.^13^

 The World Bank’s income classification for the fiscal year 2024 was used to determine LLMIC status.^18^

###  Search Strategy and Eligibility Criteria

 Literature was searched using the Embase, MEDLINE, and Scopus electronic databases in June 2024. Four domains of search terms were used: (1) partnerships (partnership, public private partnership, public-private sector partnership, PPP, public private engagement, health partnerships, public sector, private sector); (2) LMIC, using the Cochrane LMIC filter for Embase and PubMed, and related terms for Scopus (low middle income country, LMIC); (3) primary health care (primary health care, PHC, health care delivery, health care, service delivery, universal healthcare, public health, health service, health systems strengthening, health sector); and (4) governance (governance, accountability, success factor, organisational relationship).

 The search was limited to articles that related to humans, were available in English, and were published between 2000 and 2023 inclusive. We conducted searches from 2000 because PPP in health were increasingly published around this time,^10^ as was literature on governance in health systems.^13^

###  Selection Process 

 Screening of records was conducted in two phases. First, titles and abstracts were reviewed, and records were retained if they provided details about factors that enabled or constrained the governance of a PPP for the delivery of a PHC program in an LLMIC. Retained records were then read in full and inclusion and exclusion criteria applied. The inclusion criteria were a qualitative or mixed-methods study that discussed elements of local partnership implementation and governance. We chose to include articles that reported qualitative studies because we were seeking implementation experiences of partnership governance. The exclusion criteria were letters to the editor, an opinion piece, a systematic or scoping review, policy analysis, clinical trial, or a conference paper. Records were also excluded if the partnership discussed was a global health initiative, a partnership for research, or focused on the financing of a PPP; these partnerships were considered out of scope because the focus of this literature review was locally implemented PPPs for PHC. Also, articles were excluded if they lacked detail about the PPP governance or if a PPP was a recommendation for further research.

 Records identified through the database searches were supplemented by those collected from a non-systematic search using Google Scholar. All screening was conducted by GD. Questions about the inclusion of an article was discussed with AKH and AC, and consensus reached. Microsoft Excel and EndNote X8 were used to manage records and citations.

###  Data Extraction and Analysis

 The following information was extracted from each included article: author, year of publication, setting (ie, country, district within a country), World Bank income classification, PHC topic area, key stakeholders in the PPP, and information relevant to each domain of the transparency, accountability, participation, integrity, and policy capacity (TAPIC) governance framework.^19,20^ Where an article discussed multiple PPPs, only data related to PPP operating in LLMICs were extracted.

 Data were analysed thematically using a deductive approach.^21,22^ that involved coding data against a framework and then inductively identifying common themes. The results were synthesised and presented as a theme-based narrative. We used Greer and colleagues’ governance framework, with its five domains of TAPIC, which provides an evidence-based framework for assessing governance in service delivery.^19,20^ While governance is context specific we sought to identify practical mechanisms that can be adapted to help improve the governance of locally-implemented PPP for PHC in LLMIC.^20^ GD undertook data extraction and coding.

###  Quality Assessment

 The Critical Appraisal Skills Programme (CASP) qualitative checklist was used to assess the quality of articles.^23^ All articles included in this review referenced existing literature and articulated the novel contribution they made to knowledge. There was broad heterogeneity in the study designs, with 12 case studies. Two papers did not report the study design. The CASP Qualitative Checklist is provided in Supplementary file 2.

## Results

 A total of 4290 unique records were identified that were reduced to 38 articles after first stage screening. One of these articles was not available, and a further 23 articles were removed during full text review, with a total of 14 articles retained for analysis (Figure). No articles were excluded for reasons of poor quality. A summary of included articles is provided in Table. Of the 14 included articles, nine reported on PPPs in Africa: Uganda,^24^ Ghana,^25,26^ and Tanzania^27-32^; three in Asia: Cambodia^33,34^ and India^35,36^; and two in Papua New Guinea.^37,38^

**Figure:**
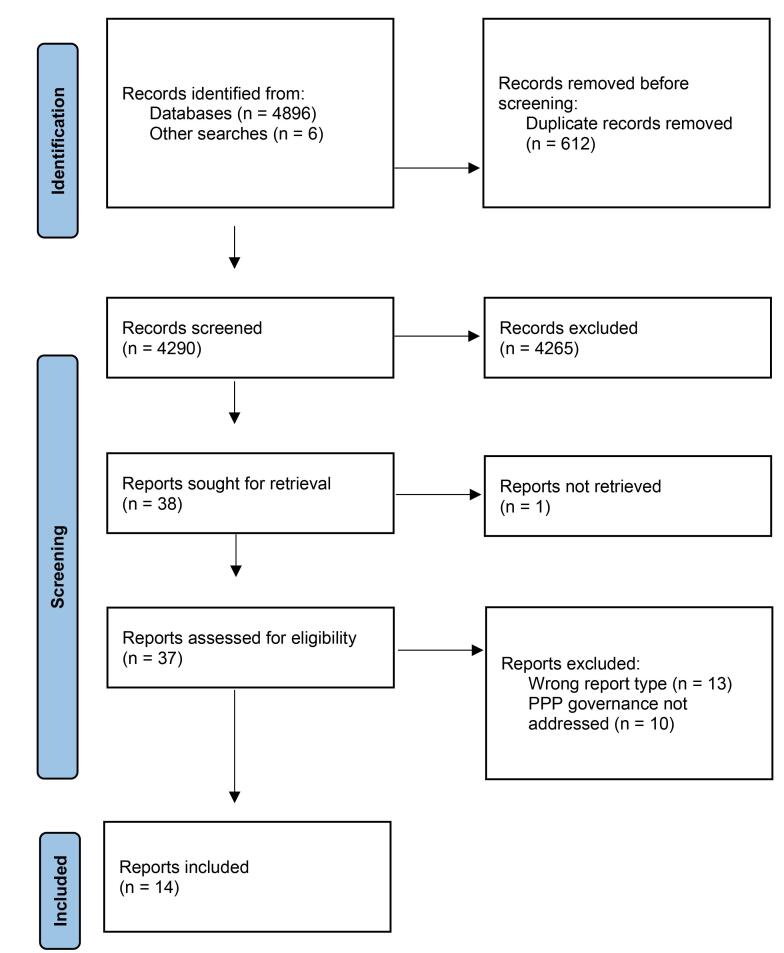
PRISMA Flow Chart of Study Selection. Abbreviation: PRISMA, Preferred Reporting Items for Systematic Reviews and Meta-Analyses.

**Table:** Key Features of Studies

**Author/s**	**Year**	**Country/ies**	**World Bank Income Classification**	**Principal PHC Topic**	**Key Stakeholders in PPP**
Amo-Adjei^25^	2016	Ghana	Lower-middle	Tuberculosis	Public service, private tuberculosis control service providers
Aveling and Martin^33^	2013	Cambodia (United Kingdom)	Lower-middle (high)	Reproductive & child health	International NGO, local NGOs, Ministry of Defence, donor funding organisation
Awale et al^35^	2019	India	Lower-middle	Polio	International and local NGOs, country secretariat, Ministry of Health, UNICEF, Rotary, donor funding organisations
Hushie^26^	2016	Ghana	Lower-middle	Maternal and child health, HIV/AIDS, reproductive health, eye health, mental health	Five case studies: Government organisations, NGOs, donor funding organisations
Kamugumya and Olivier^27^	2016	Tanzania	Lower-middle	Reproductive & child health	District government organisations, NGOs, private providers
Miles et al^37^	2014	Papua New Guinea	Lower-middle	HIV	National and provincial governments, contracted implementation organisation, donor funding organisations, beneficiary communities
Mkoka et al^28^	2014	Tanzania	Lower-middle	Reproductive & child health	District government (Council Health Management Teams), NGOs, donor funding and development partners, health facilities, beneficiary communities
Mshana et al^29^	2018	Tanzania	Lower-middle	PHC	National, regional and local government, private sector service providers
Njau et al^30^	2009	Tanzania (Other)	Lower-middle (Unspecified)	Malaria	National level NGOs
Nuhu et al^31^	2020	Tanzania	Lower-middle	PHC	National and district governments, public district health facilities, private district health facilities
Orobaton et al^24^	2007	Uganda	Low	PHC	District/local governments, civil society organisations, contracted management organisation, donor funding organisation
Prasad et al^32^	2022	Tanzania	Lower-middle	Reproductive & child health	National, regional, district governments, evaluation organisation; technical (obstetric, reproductive health) organisations; donor funding organisations
Salve et al^36^	2018	India	Lower-middle	Tuberculosis	National and district governments, NGOs, private health service providers
Thomason and Rodney^38^	2009	Papua New Guinea	Lower-middle	PHC	National, provincial and district governments, private sector companies (as donor funding organisations), contracted implementation organisations

Abbreviations: PPP, public-private partnership; PHC, primary healthcare; NGO, non-governmental organisation; UNICEF, United Nations Children’s Fund.

 By World Bank economic classification, one article reported on a PPP operating in a low-income country (Uganda),^24^ while 11 articles reported on PPPs in lower-middle-income countries (Ghana,^25,26^ Cambodia,^33^ India,^35,36^ Tanzania,^27-32^ and Papua New Guinea^37,38^). Two articles reported on PPPs in a lower-middle income country and one other country; the other country was a high-income country in one article and an unspecified country in the other.^30,33^

 Four of the articles reported on PPPs for delivery of generalist PHC services^24,29,31,38^; five focused on the delivery of disease-specific interventions at the PHC level (two for tuberculosis control,^25,36^ one for HIV,^37^ one for malaria,^30^ and one for polio^35^); and five for delivery of a reproductive and child health program.^26-28,32,33^

 Our thematic analysis used the five domains of the TAPIC framework: transparency, accountability, participation, integrity, and policy capacity. These domains are discussed in the following sub-sections.

###  Transparency

 In the TAPIC framework, the transparency domain is concerned with informing stakeholders of decisions made or being made and the decision-making processes.^19,20^ Mechanisms for strengthening transparency identified in the framework include committees, reporting, performance reporting and assessment; and importantly, the result of the right levels of transparency is trust.^19^

 In the articles we reviewed, constant communication between partners was emphasised as an enabler of governance, as it facilitated transparency through information sharing and teamwork.^26,29,36^ Communication had broader implications than solely a means of information sharing. It was reported to have led to trust-building among partners and assisted in fostering and facilitating ownership of the PPP.^32^ Joint work planning enabled transparency in resource allocation and reporting,^24^ as did ensuring clarity of the partnership structure for all stakeholders.^35^

 In contrast, a lack of strategic communication and insufficient consultation and communication^29,31^ were explicitly highlighted in the articles reviewed as barriers to transparency because their absence discouraged or disallowed joint planning and decision-making. Weak transparency was identified in the articles with examples including a lack of clarity on resource allocation,^25^ delays in receiving allocated funds through the partnership^30,31^ and top-down and one-way information sharing or decision-making.^27,31,33^

###  Accountability

 Accountability in the TAPIC framework focuses on stakeholders accounting for their actions to those who can take appropriate action and administer consequences. In a practical sense, accountability mechanisms identified in the framework can include contracts, pay for performance, standards, and codes of conduct.^19,20^

 Eleven of the 14 articles discussed formal agreements as accountability mechanisms, emphasising the value of having a memorandum of understanding (MOU),^24-27,30,35-37^ or a contract^31^ in place between the primary partners. These examples of formal agreements between partners were noted as critical to the PPP because they explicitly defined the partners’ roles and responsibilities, provided a clear goal for the partnership, as well as providing policy direction. In instances where funding was linked to an MOU and MOU renewals were delayed, there was uncertainty and potential for distrust among stakeholders.^36^

 A similar accountability mechanism identified in the articles was the use of a third party or contracted partner to assist in managing and maintaining relationships.^37,38^ The value was that they were able to manage differences between partners and assist with maintaining the partnership’s focus.^38^ A final mechanism identified was performance-based funding with annual targets and while this mechanism provided opportunity for monitoring against clear indicators authors reported that partners felt it diverted attention from implementation to administration.^33^

###  Participation

 The third domain in the TAPIC framework, participation, is focused on ensuring that the stakeholders affected by a decision can express their views. Examples of participation mechanisms include stakeholder forums, consultations, advisory committees, elections, and surveys.^19,20^

 Participation mechanisms were discussed in 13 of the articles and addressed issues that facilitated engagement such as developing relationships, collaborative activities, and communication. Actions that promoted and enabled the development of valuable relationships were identified as early engagement between partners, and spending time building relationships and respect between all partners.^26,33,36,37^

 Three articles discussed the presence of a representative body of partner organisations and partners, where each stakeholder had their own technical capacity. In the reviewed articles, this representative body included a range of stakeholders at all levels of partner organisations,^24^ including all levels of government (national, regional, and district), donors, political leaders, technical partners, and communities, where applicable,^28,36^ and the use of stakeholder meetings to review progress.^26^ Similar to the use of a third-party contractor identified in the accountability domain above, an individual’s engagement as a champion of the partnership helped to strengthen and formalise relationships and participation.^30^

 In the reviewed articles, specific constraints to participation were identified, such as declining engagement of stakeholders, lack of autonomy,^25^ misalignment between partners, lack of trust, reinforced boundaries, and resistance from staff.^31^ Noted in six articles^25,27,30,33,37,38^ the issue of power imbalance within relationships was a constraint to effective relationships and participation. For example, Amo-Adjei identified that actions such as bias towards public sector partners who received more resources was an expression of power that negatively impacted relationships; and therefore, participation.^25^ It was acknowledged that PPPs could perpetuate power imbalances through hierarchies within the partnership and through existing dynamics when partners may have had prior relationships.^30,33^

###  Integrity

 The fourth domain of the TAPIC framework is integrity which is focused on having clear roles and responsibilities and clear related processes.^19,20^ Integrity mechanisms identified in the framework include procedures, internal and external audit, clear individual and organisational roles, and budgets.^19,20^

 Integrity-strengthening mechanisms identified in the articles were the use of standard operating procedures which provided clear processes and assisted in risk management,^37^ internal audits and reviews,^26^ and the incorporation of technical oversight into the partnership’s design through the establishment of or use of technical advisory groups^30,32,35^ or technical programs.^25^ Technical oversight was discussed in articles that presented partnerships for disease-specific and reproductive and child health programs, rather than those for generalist PHC. The value of technical oversight to PPP governance was that it built rigour and contributed to clarity of roles and responsibilities within the PPP.

 The importance of clear roles and responsibilities was identified in all articles. For example, the presence of clear roles and responsibilities led to defined and discrete contributions from partners,^35^ shared understanding of the partnership,^24,32,35,37^ and clear linkages between partners.^28^ In contrast, when roles and responsibilities were not clear, authors reported that it made managing partner expectations difficult,^25^ and led to confusion in the implementation of partnership activities.^29^

###  Policy Capacity

 The final domain of the TAPIC framework is policy capacity, where there is ability to develop policy that is aligned with resources and purpose. It has also been explained as seeking research and using acquired knowledge.^20^ The mechanisms identified in the framework that can improve capacity—as relevant to this study—include monitoring and evaluating, and intelligence on performance and processes.^19,20^

 Capacity mechanisms identified in the reviewed articles included establishing key performance indicators and monitoring processes.^26,31,35,37^ PPPs with monitoring and evaluation requirements embedded within the design reported effective oversight of the PPP. This was highlighted by Awale et al who noted that “field-based learning and continuous analysis of inputs, processes, and outputs were responsible for the emergence of such strong partnerships”^35^ and “used monitoring data and took observations of the monitors very seriously.”^35^

 In the reviewed articles, information was collated, shared, and used in various ways across the PPP. Examples included information that was gathered and shared regularly was used to direct and improve the PPP and its activities^26,32^; and incorporating knowledge and information local to the LLMIC context In the absence of formal knowledge sharing processes, the informal use of information, for example, to adapt the program to the local context, was considered beneficial to the PPP and helped to facilitate trust and accountability within the overall governance.^27,33^

 A final aspect of relevance to the capacity domain in the present climate of development and donor funding is localisation, which was described in three articles.^24,32,33^ One enabling factor discussed in the articles was employing staff locally for the partnership Prasad et al described the which had a positive impact on the partnership through fostering local collaboration and knowledge sharing.^32^ The program manager was employed part-way through implementation, and the authors acknowledged that this should have occurred earlier.^32^ This was supported by Aveling and Martin’s article, where locally employed staff spoke local languages, leading to stronger relationships between partners.^33^ A second enabler discussed was locating the staff at the site of partnership activities which had multiple benefits, including proximity to partners for relationship-building,^33^ ability to spend time conducting activities and delivering services,^32,33^ and a greater understanding of local context enabling the partnership to address local needs and respond promptly.^24^ These factors related to localisation were present in articles covering three different locations; Tanzania,^32^ Cambodia,^33^ and Uganda,^24^ and presented in generalist PHC^24^ and reproductive and child health^32,33^ partnerships, which highlights that it is an important consideration across contexts and settings.

## Discussion

 Fourteen articles were included and reviewed to synthesise knowledge about the factors that enable and constrain the governance of locally implemented PPPs for PHC in LLMICs. We used the TAPIC framework and its five domains (transparency, accountability, participation, integrity, and policy capacity) to structure our analysis.^19,20^ In identifying the domains, consideration was given to whether they enabled or constrained governance in the PPP. While the TAPIC framework emphasises the importance of context, we found common enabling factors across the articles which we now discuss, along with suggestions for practice.

 Within the Transparency domain of the TAPIC framework, resources were a dominant theme. While insufficient resources are not considered a governance problem,^20^ our review identified that unequal and top-down resource allocation, opaque and poorly communicated resource allocation were barriers to effective governance in the reviewed PPPs. The allocation ofadequate resources should be incorporated into the design and governance of a partnership. Ideally, the roles and responsibilities set out in an MOU would cover the resources each partner brings to the partnership. Likewise, the processes for collaborative and transparent allocation of those resources should be detailed in the MOU or partnership guidelines. If the practicalities of working together are clear and can be addressed early, then more time and effort can be dedicated to other elements of the partnership.

 Relevant to the domains of Accountability and policy Capability, monitoring and evaluation was a consideration highlighted in our review. Monitoring and evaluation is a tangible action that PPP partners can incorporate into the PPP. Accountability could be addressed and incorporated by building in agreed targets, processes, and responsibilities; establishing a monitoring and evaluation system; and using the data generated for reviews, planning and decision-making. In practice, these elements of accountability could be discussed by all partners at the inception and design phase of partnership and scheduled for regular reviews throughout implementation.

 In relation to the Participation domain, the nature of engagement between partners was found to be an enabling factor. This included developing relationships, collaborative activities, ensuring genuine representation, and open communication. This finding is important because adequate time and opportunity are required to develop meaningful and collaborative relationships, particularly in an LLMIC setting where partners in a PPP are more likely to come from diverse backgrounds and have different cultural and communication norms. Partnerships can facilitate increased understanding through—for instance—ensuring early and constant communication, engaging staff from the country or location in senior stakeholder engagement and liaison roles, having a presence in the program or activity locations, and convening regular meetings in each organisation’s premises.

 Finally, within the Integrity domain, our review identified that incorporating shared goals and formal agreements between partners, clear roles and responsibilities, and sufficient policy direction, enables effective governance by providing the PPP and its members with an overall structure within which to set direction and plan actions. This finding can be applied by PPP practitioners by considering the LLMIC context and partners in the PPP. While it would ideally be actioned early in the development of the PPP, these factors can be established, or reviewed, during PPP implementation.

 In an LLMIC context, financial and human resources for health can be scarce and competitive, affecting relationships, dynamics, collaborative actions, and PPP performance. This means the strength of the existing health system and its capacity to support a PPP will need to be integral to the PPP’s design, as will examining the role of external stakeholders that bring financial and accountability requirements to the PPP. The TAPIC framework’s authors emphasise that the domains and the corresponding mechanisms are not a checklist for governance^20^; rather, the framework provides structure to consider operating dynamics and, building on this, develop strategies to enhance partnership’s performance.

 Our review identifies some knowledge gaps that additional research may address. These relate to further evidence that could be found in unpublished literature such as evaluation reports of locally implemented PPPs and conducting in-depth case studies of local implementation experiences. A comparison of evidence from high-ncome countries with LLMIC would extend our findings. These are just three opportunities for future research in this area.

 This work is not without limitations. First, the review is limited to experiences documented in the peer-reviewed literature, likely a sample of learning about PPPs for PHC. Second, most articles had a case study design, leading to a broad heterogeneity of articles and a limited ability to compare outcomes. Third, the authors recognise that partnerships and their governance are dynamic and nuanced and that factors contributing to the success of PPP may not be captured in the published literature. Finally, we acknowledge that a selection bias may have been introduced given one author conducted the searches and data extraction. Despite these limitations, the study is valuable as it is the first to systematically collect and present evidence on the enablers and constraints of governance in locally implemented PPPs for the delivery of PHC in LLMIC.

## Conclusion

 This review synthesises literature related to the enablers and barriers to the governance of locally implemented PPP for PHC in LLMICs over the past 24 years. The five domains of the TAPIC framework, applied to 14 articles, provide practical guidance for considering governance in PPP. Context is a critical feature of the TAPIC framework and of locally implemented PPP for PHC, and thus the framework provides valuable opportunities for considering and improving governance in these implementation settings. Despite contextual differences in the PPPs reviewed, we found four themes that enable governance: the importance of PPP design, stakeholder engagement and participation, resource allocation, and monitoring and evaluation. These common themes help facilitate the successful implementation of a PPP and thus influence the PPP’s impact on health outcomes, through establishing and maintaining healthy working relationships between partners, and defining and documenting systems and processes. Despite some limitations this review provides valuable insights on enablers and constraints of governance in PPP for PHC that are implemented at a local level. Further research on this topic in LLMIC, and a comparison with high-ncome countries, would expand the understanding of governance in locally implemented PPP for PHC.

## Ethical issues

 Not applicable.

## Conflicts of interest

 Authors declare that they have no conflicts of interest.

## Supplementary files


Supplementary file 1. PRISMA Checklist.

Supplementary file 2. Results of CASP Qualitative Checklist.
