# Supplementary material for: Governance of Public-Private Partnerships for Primary Healthcare in Low- and Lower-Middle-Income Countries, 2000-2023: A Systematic Review
Source: Int J Health Policy Manag. 2025 Mar 8;14:8442. doi: 10.34172/ijhpm.8442 (PMC12032253; doi:10.34172/ijhpm.8442)
Supplement: Supplementary file 2 — Results of CASP Qualitative Checklist. [file ijhpm-14-8442-s002.pdf]

Article title: Governance of Public-Private Partnerships for Primary Healthcare in Low- and Lower-Middle-Income Countries, 2000-2023: A Systematic Review

Journal name: International Journal of Health Policy and Management (IJHPM)

Authors' information: Georgina Dove[1]*, Adam Craig[2], Ben Harris-Roxas[1], Angela Kelly-Hanku[3,1]

[1]University of New South Wales, Sydney, NSW, Australia.

[2]University of Queensland, Brisbane, QLD, Australia.

[3]Papua New Guinea Institute of Medical Research, Goroka, Papua New Guinea.

***Correspondence to:** Georgina Dove; Email: g.dove@student.unsw.edu.au

Citation: Dove G, Craig A, Harris-Roxas B, Kelly-Hanku A. Governance of public-private partnerships for primary healthcare in low- and lower-middle-income countries, 2000-2023: a systematic review. Int J Health Policy Manag. 2025;14:8442. doi:10.34172/ijhpm.8442

Supplementary file 2. Results of CASP Qualitative Checklist

Amo-Adjei (2016)	Aveling et al (2013)	Awale et al (2019)	Hushie (2016)	Kamugumya et al (2016)	Miles et al (2014)	Mkoka et al (2014)	Mshana et al (2018)	Njau et al (2009)	Nuhu et al (2020)	Orobaton et al (2007)	Prasad et al (2022)	Salve et al (2018)	Thomason et al (2009)
**A. Are the results valid:**													
1. Was there a statement of the aims of the research?													
Y	Y	Y	Y	Y	Y	Y	Y	Y	Y	Y	Y	Y	Y
2. Is a qualitative method appropriate?													
Y	Y	Y	Y	Y	Y	Y	Y	Y	Y	Y	Y	Y	Y
3. Is it worth continuing?													
Y	Y	Y	Y	Y	Y	Y	Y	Y	Y	Y	Y	Y	?
4. Was the recruitment strategy appropriate to the aims of the research?													
Y	N/A	N/A	Y	Y	Y	Y	Y	Y	Y	N/A	N/A	Y	N/A
5. Was the data collected in a way that addressed the research issue?													
Y	Y	Y	Y	Y	Y	Y	Y	Y	Y	Y	Y	Y	N/A
6. Has the relationship between researcher and participants been adequately considered?													
Y	Y	Y	Y	Y	Y	Y	?	Y	Y	?	?	Y	Y
**B. What are the results:**													
7. Have ethical issues been taken into consideration?													
Y	Y	Y	Y	Y	N/A	Y	N/A	N/A	Y	?	?	Y	N
8. Was the data analysis sufficiently rigorous?													
Y	Y	Y	Y	Y	?	Y	Y	Y	Y			Y	N
9. Is there a clear statement of findings?													
Y	Y	Y	Y	Y	Y	Y	Y	Y	Y	Y	Y	Y	Y
**C. Will the results help locally:**													
10. How valuable is the research?													
H	H	H	H	H	H	H	M	M	M	H	H	M	H

Note: Y = reported, N = not reported, ? = can't tell, N/A = not applicable, H = high value, M = moderate value, L = low value
